# Impact of Young Age on the Prognosis for Oral Cancer: A Population-Based Study in Taiwan

**DOI:** 10.1371/journal.pone.0075855

**Published:** 2013-09-26

**Authors:** Ting-Shou Chang, Chun-Ming Chang, Hsu-Chieh Ho, Yu-Chieh Su, Li-Fu Chen, Pesus Chou, Ching-Chih Lee

**Affiliations:** 1 Department of Otolaryngology, Kaohsiung Veterans General Hospital, Kaohsiung, Taiwan; 2 Department of Surgery, Buddhist Dalin Tzu Chi General Hospital, Chiayi, Taiwan; 3 Community Medicine Research Center and Institute of Public Health, National Yang-Ming University, Taipei, Taiwan; 4 Department of Otolaryngology, Buddhist Dalin Tzu Chi General Hospital, Chiayi, Taiwan; 5 Department of Internal Medicine, Buddhist Dalin Tzu Chi General Hospital, Chiayi, Taiwan; 6 School of Medicine, Tzu Chi University, Hualian, Taiwan; 7 Department of emergency, National Yang-Ming University Hospital, Taipei, Taiwan; Johns Hopkins University, United States of America

## Abstract

**Background:**

Oral cancer leads to a considerable use of health care resources. Wide resection of the tumor and reconstruction with a pedicle flap/ free flap is widely used. This study was conducted to investigate if young age at the time of diagnosis of oral cancer requiring this treatment confers a worse prognosis.

**Methods:**

A total of 2339 patients who underwent resections for oral cancer from 2004 to 2005 were identified from The Taiwan National Health Insurance Research Database. Survival analysis, Cox proportional regression model, propensity scores, and sensitivity test were used to evaluate the association between 5-year survival rates and age.

**Results:**

In the Cox proportional regression model, the older age group (>65 years) had the worst survival rate (hazard ratio [HR], 1.80; 95% confidence interval [CI], 1.45-2.22; P<0.001). When analyzed using the propensity scores, the adjusted 5-year survival rates were also poorer for oral cancer patients with older age (>65 years), compared to those with younger age (<45 years) (P<0.001). In sensitivity test, the adjusted hazard ratio remained no statistically elevated in the younger age group (<45 years).

**Conclusions:**

For those oral cancer patients who underwent wide excision and reconstruction, young age did not confer a worse prognosis using a Cox proportional regression model, propensity scores or sensitivity test. Young oral cancer patients may be treated using general guidelines and do not require more aggressive treatment.

## Introduction

Oral cancer is among the ten most common forms of cancer in the world [1]. Oral cancer is much more predominant in Taiwanese males than in females and the prevalence in male peaks at age between 45 and 65 years old [2,3]. These patients have a high incidence of tobacco use, alcohol abuse, and betel nut chewing. A trend of rising incidence has been noted on a global scale irrespective of whether the examination is of Western countries or Asian countries such as Taiwan [3,4]. The increasing economic burden of oral cancer treatment has become obvious. Of all cancers in males in Taiwan, oral cancer had been ranked fourth in incidence and mortality since 1995. Up to $1195 million (in U.S. dollars) was spent on the treatment of oral cancer in 2004. As most countries, only a small percentage (0.4-3.6%) of these lesions occurred in patients younger than 45 years old. However, the number of young patients with oral cancer is increasing. Oral cancer is now a serious socioeconomic problem as well as an important public health issue in Taiwan.

Studies about whether age at diagnosis affects prognosis have produced conflicting data. Son and Kapp [5], Amsterdam and Strawitz [6] and Sakanria and Harari [7] concluded that young patients had a worse outcome than their older counterparts. Fridllander et al. [8], Pitman et al. [9], Vargas et al. [10], Glory et al. [11] and Pytynia et al. [12] noted that there were no significant differences in outcome between the different age groups. However, McGrefor et al. [13], Clark RM et al. [14], Hafkamp et al. [15], Carniol and Fried [16], and Lacy PD et al. [17] all showed that the prognosis for young patients was better. Because of the disparity in the results of these studies, the question of outcome between different age groups remains unanswered. The number of cases in these studies was small, and the power of each study was therefore in question.

We designed a population-based analysis between young patients and older patients with oral cancer in order to address the issue of outcome. The purpose of this study was to examine the relationship between different age groups and survival rates using a population-based database for patients following resection of oral cancer with reconstruction.

## Materials and Methods

### Ethics statement

This study was initiated after being approved by the Institutional Review Board of Buddhist Dalin Tzu Chi General Hospital, Taiwan. Because the identification numbers and personal information of the individuals included in the study were not included in the secondary files, the review board stated that written consent from patients was not required.

### Database

The data for this study were collected from Taiwan’s NHIRD for the years 2004 to 2008. This dataset is organized and managed by Taiwan’s National Health Research Institutes but collected by Taiwan’s National Health Insurance Program, which has been in place in Taiwan since 1995. The program covers approximately 99% of the residents in Taiwan and has contracts with 97% of the medical providers there [18]. To verify accuracy of diagnosis, Taiwan’s Bureau of National Health Insurance randomly reviews the charts of one per 100 ambulatory and one per 20 inpatient claims and interviews patients [19,20]. The reliability of the database for the research was admitted in the world [21,22]. Due to the protection of personal confidential data, cancer stage and some risk factors (e.g., smoking status, alcohol drinking, betel nut chewing) could not be linked to primary survey data and were not included in this dataset.

Our study cohort consisted of Taiwan’s incidental oral cancer patients (*International Classification of Diseases, Ninth Revision, Clinical Modification* [ICD-9-CM] codes 140-145, excluding 142, salivary gland cancer) who had received wide excision and free-flap or pedicle-flap reconstruction with or without adjuvant therapy between 2004 and 2005. Survival of each oral cancer patient was determined by linking their 2004 to 2008 mortality data extracted from catastrophic files for first curative treatment up to 5 years prior to death. With these data, we could calculate death-free survival.

### Measurements

The key dependent variable of interest was 5-year overall survival rate. The use of overall survival data should not interfere significantly with our results because, as Roohan et al. have shown in a study adapting a clinical morbidity index for use with ICD-9-CM administrative databases, there is no significant difference between survival models for all-cause-mortality and cancer-specific mortality [23].

The key independent variable was age, which was sorted into three groups (<45 years, 45-65 years, and >65 years). Patient characteristics included gender, geographic location, treatment modality, severity of disease, tumor site, and individual socioeconomic status. The disease severity for each patient was based on the Charlson Comorbidity Index score, which is widely used for risk adjustment in administrative claims data sets. We used a modified Charlson Comorbidity Index score calculated as the sum of weighted scores based on the relative mortality risk for 19 conditions [24].

This study used enrollee category (EC) as a proxy measure of socioeconomic status, an important prognostic factor for cancer. This classified the oral cancer patients into 4 subgroups: EC 1 (civil servants, full-time or regular paid personnel with a government affiliation), EC 2 (employees of privately owned institutions), EC 3(self-employed individuals, other employees, and members of the farmers’ or fishermen’s associations), EC 4 (veterans, low-income families, and substitute service draftees) [25]. The level of urbanization was determined by population density, percentage of residents with college or higher education, percentages of residents over 65, percentage of residents who were agriculture workers, and the number of physicians per 100,000 people [26]. We recorded the level of urbanization as urban (urbanization level 1), sub-urban (urbanization level 2-3) or rural (urbanization 4-7).

### Statistical analysis

All statistical operations were performed using SPSS (version 15, SPSS Inc., Chicago, IL, USA). Pearson’s chi-square test was used to explore the differences between categorical variables in the different age groups. Continuous variables were analyzed by one-way ANOVA.

The cumulative 5-year survival rates and the survival curves were constructed and compared by the log-rank test. Survival was measured from the time of oral cancer resection by using overall death as censoring variables. The Cox proportional regression model and the survival analysis with propensity score stratification were used to compare outcomes between different age groups.

#### (1): Cox proportional hazards model

The Cox proportional regression model was used to evaluate the age effect on oral cancer survival rates after adjusting for demographic variables, hospital characteristics and treatment modalities.

#### (2): Propensity score

Propensity score stratification was applied to replace the wide host of confounding factors that may be present in an observational study with a variable of these factors [27-30]. To derive the propensity score in this study, patient characteristics were entered into a logistic regression model predicting selection for different category of the age groups. The characteristics included the year gender, the Charlson Comorbidity Index score, individual SES, geographic area and urbanization of residence, tumor site and treatment modality, provider caseload, and hospital characteristics. The effect of age on the 5-year survival rate was analyzed within each quintile. The Mantel-Haenszel odds ratio was calculated in addition performing the Cochran-Mantel-Haenszelχ^2^ test.

#### (3): Sensitivity test

Up to 99% hospitals in Taiwan were enrolled in the program of Taiwanese cancer data register conducting by the Bureau of Health Promotion, Department of Health. We used data from its database of oral cancer staging by the American Joint Committee on Cancer (AJCC) staging classification to identify the stage distribution of oral cancer in Taiwan [31]. Up to 83% of operated oral cancer patients had early stage disease. Besides, near 77% of oral cancer patients who underwent surgery with adjuvant therapy had advanced stage disease. Due to lack of cancer stage in our NHIRD database, we did two cancer stage simulation models to evaluate the survival after adjusting estimated oral cancer stage distribution in different groups of treatment (Appendix S1 and Appendix S2).

## Results

The mean age of oral cancer patients was 53±11. The median follow-up time was 42 months (range, 2-60 months). Table 1 shows patient characteristics. Almost forty-six percent patients underwent surgery followed by adjuvant therapy. The youngest group (age<45) were more likely to be male, have tongue cancer, receive adjuvant therapy and possess a better SES. The oldest group (age>65) was associated with female gender and rural residential area (*P*<0.001).

**Table 1:** Demographic characteristics for oral cancer patients (*n*=2339).

	Age<45				Age 45-65			Age>65		*P* value
	(*n*=608)				(*n*=1416)			(*n*=315)		
	n		(%)		n	(%)		n	(%)	
Age (mean ±SD)	39.58±4.30				54.11±5.45			71.37±4.93		<0.001
Gender										<0.001
Male	591		(97)		1350	(95)		278	(88)	
Female	17		(3)		66	(5)		37	(12)	
Primary site										<0.001
Tongue	159		(26)		241	(17)		32	(10)	
Buccal mucosa	279		(46)		575	(41)		134	(43)	
Others	170		(28)		600	(42)		149	(47)	
CCIS group										0.120
≦4	391		(64)		972	(69)		219	(70)	
>4	217		(36)		444	(31)		96	(30)	
Treatment modality										0.009
Surgery	304		(50)		777	(55)		190	(60)	
Surgery +adjuvant therapy	304		(50)		639	(45)		125	(40)	
Surgeon caseload within two years										0.665
Low (1-25)	298		(49)		668	(47)		155	(49)	
High (>25)	310		(51)		748	(53)		160	(51)	
Hospital level										0.335
Medical center	504		(83)		1163	(82)		249	(79)	
Region/district hospital	104		(17)		253	(18)		66	(21)	
Socioeconomic status										<0.001
High	212		(35)		406	(29)		71	(22)	
Medium	237		(39)		776	(55)		191	(61)	
Low	159		(26)		234	(16)		53	(17)	
Geographic region										0.568
Northern	233		(38)		517	(37)		99	(31)	
Central	108		(18)		259	(18)		65	(21)	
Southern	245		(40)		580	(41)		138	(44)	
Eastern	22		(4)		60	(4)		13	(4)	
Urbanization level										<0.001
Urban	149		(24)		337	(24)		56	(18)	
Suburban	253		(42)		689	(49)		109	(35)	
Rural	206		(34)		390	(27)		150	(48)	

CCIS, Charlson Comorbidity Index Score.

**Figure 1:**
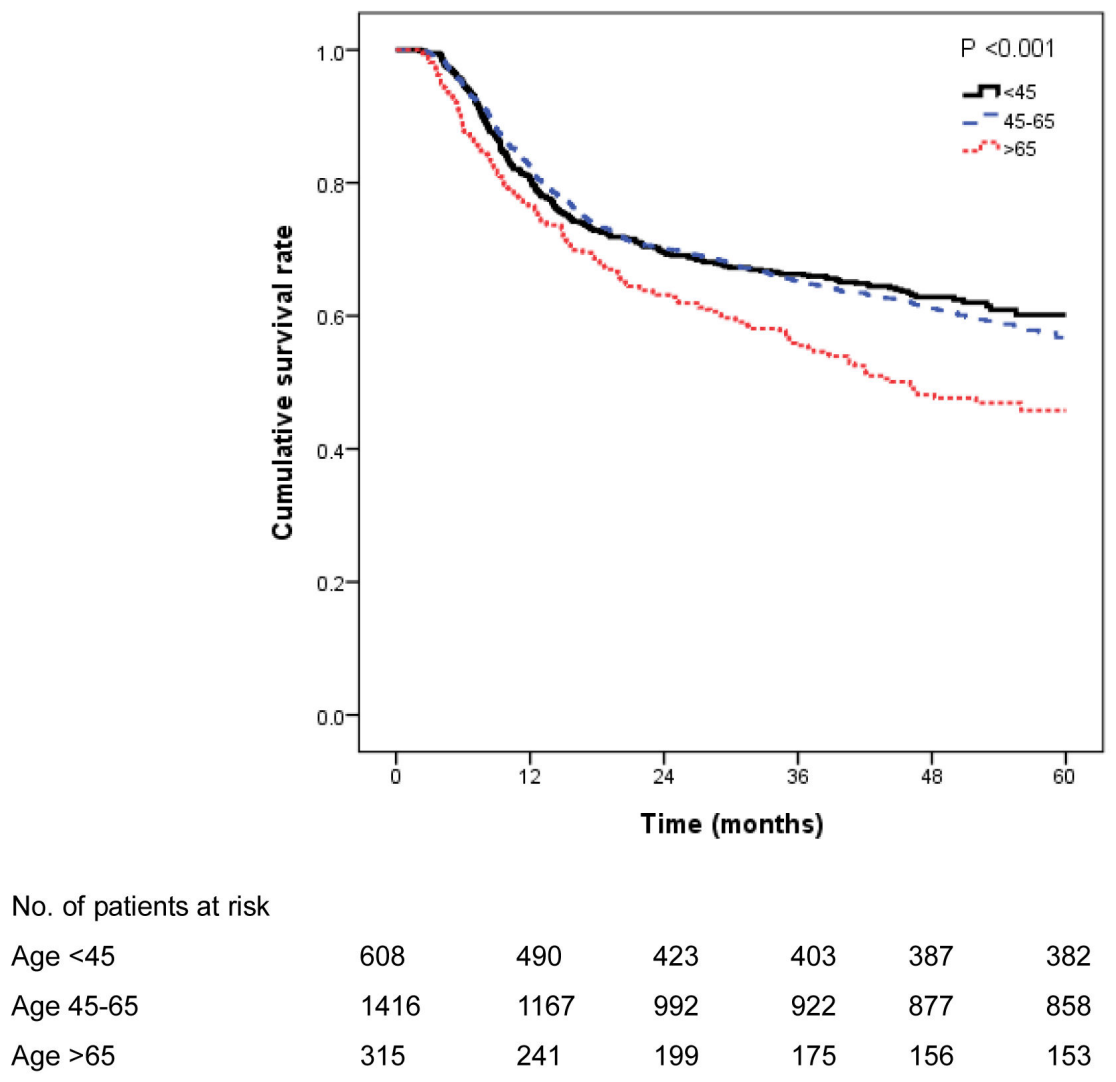
Effect of age on survival rates of patients with oral cancer (*n*=2339).

The overall survival curves are shown in Figure **1**. The 5-year survival rate for study population was 56% (95% confidence interval [CI], 54-59%): 60.1% (95% CI, 56-65%) for the youngest group, 56.7% (95% CI, 53-60%) for the middle-aged group, and 45.8% (95% CI, 40-52%) for the oldest group. The oldest group had the worst prognosis (*P*=0.001).

Table **2** shows the details of the adjusted hazard ratios based on the Cox proportional hazards regression model. After adjustment for the patients’ gender, primary tumor site, Charlson Comrobidity Index score, treatment modality, surgeon caseload, hospital teaching level, enrollee category, level of urbanization, and geographic region, the hazard ratio for death was 1.80-times (95% CI, 1.45-2.22; *P*<0.001) greater for the oldest group than for the youngest group. After adjusting for other factors, patients with an increased Charlson Comorbidity Index score, residing in the central and eastern geographic region, and receiving adjuvant therapy had a greater likelihood of death.

**Table 2:** Adjusted hazard ratios for different age groups (*n*=2339).

	Hazard ratio	95% CI	*P* value
Age group			
<45y/o	1		
45-65 y/o	1.18	1.01-1.38	0.040
>65y/o	1.80	1.45-2.22	<0.001
Male	1.12	0.84-1.49	0.443
Primary site			
Tongue	1		
Buccal mucosa	0.79	0.67-0.94	0.008
Others	0.81	0.68-0.96	0.015
CCIS group			
≦4	1		
>4	1.94	1.70-2.22	<0.001
Treatment modality			
Surgery	1		
Surgery +adjuvant therapy	2.23	1.94-2.56	<0.001
Surgeon caseload with two years			
Low (1-25)	1		
High (>25)	0.78	0.68-0.90	<0.001
Hospital level			
Medical center	1		
Region/district hospital	1.05	0.88-1.25	0.603
Socioeconomic status			
High	1		
Medium	1.01	0.86-1.18	0.896
Low	1.19	0.98-1.43	0.078
Geographic region			
Northern	1		
Central	1.28	1.05-1.55	0.014
Southern	1.11	0.95-1.30	0.203
Eastern	1.51	1.08-2.13	0.018
Urbanization level			
Urban	1		
Suburban	0.97	0.81-1.15	0.693
Rural	0.99	0.81-1.21	0.918

95% CI, 95% confidence interval; CCIS, Charlson Comorbidity Index.

Table **3** and Table **4** reveal the survival rates for different age groups after propensity score stratification (<45 years vs. age 45-65, and <45 years vs. >65 years). In Table 3, there was no difference on 5-year survival rates between young age (<45 years) and middle-age group (45-65 years). In Table 4, patients with older age (>65 years) had lower 5-year survival rates in most situations, compared to those with young age (<45 years). The p-value for Cochran-Mantel-Haenszel statistics comparing survival rates for young age (<45 years) and older age (>65 years), controlling for propensity scores, was <0.001. The adjusted 5-year survival rates for oral cancer patients with young age (<45 years) were higher than those with older age (>65 years).

**Table 3:** Five-year cumulative risk of mortality among the patients with different age groups (n=2024)^**a**^.

stratum	age <45 (n=608)				age 45-65 (n=1416)			P value
	No.	% of stratum	Survival rate (%)		No.	% of stratum	Survival rate (%)	
1	72	17.8	75.0		333	82.8	67.6	0.217
2	83	20.4	63.9		323	79.6	64.4	0.927
3	112	27.3	61.6		298	72.7	56.4	0.339
4	147	36.7	66.7		254	63.3	58.7	0.112
5	194	48.3	55.7		208	51.7	51.9	0.451
Total	608		64.6		1416		59.8	<0.001
								0.045^b^

a Stratum 1 had the strongest propensity for being oral cancer, aged 45-65; stratum 5, the strongest propensity for being oral cancer, aged <45.

b Conchran-Mantel-Haenszel statistics; adjusted odds ratio=0.81, 95% confidence interval=0.66-0.99

**Table 4:** Five-year cumulative risk of mortality among the patients with different age groups (n=923)^**a**^.

stratum	age <45 (n=608)				age >65 (n=315)			P value
	No.	% of stratum	Survival rate (%)		No.	% of stratum	Survival rate (%)	
1	75	40.8	77.3		109	59.2	45.9	<0.001
2	107	57.8	68.2		78	42.2	50.0	0.028
3	126	68.1	59.5		59	31.9	57.6	0.334
4	135	73.4	70.4		49	26.6	40.8	0.001
5	165	89.2	49.1		20	10.8	50.0	0.500
Total	608		64.9		315		48.9	<0.001
								<0.001^b^

a Stratum 1 had the strongest propensity for being oral cancer, aged >65; stratum 5, the strongest propensity for being oral cancer, aged <45.

b. Conchran-Mantel-Haenszel statistics; adjusted odds ratio=0.47, 95% confidence interval=0.35-0.64

Without age group stratification, each additional year of age was associated with additional 2% death risk (HR, 1.02; 95% CI, 1.01-1.02). However, during stratified analysis, age was not associated with increased death risk (Table 5).

**Table 5:** The adjusted hazard ratios for mortality for each additional year of age (n=2339).

	Unadjusted HR				Adjusted HR		
	HR	(95% CI)	P value		HR	(95% CI)	P value
All oral cancer patients	1.01	(1.00-1.02)	0.002		1.02	(1.01-1.02)	<0.001
Stratified analysis							
Oral cancer patients, age <45	1.01	(0.98-1.04)	0.652		1.00	(0.97-1.03)	0.919
Oral cancer patients, age 45-65	1.00	(0.98-1.01)	0.544		1.01	(0.99-1.02)	0.441
Oral cancer patients, age >65	1.01	(0.98-1.05)	0.473		1.01	(0.98-1.05)	0.542

HR, hazard ratio; 95% CI, 95% confidence interval

Cancer stage was not available in this database. In order to adjust the possible selection bias between different age groups, sensitivity test using simulation cancer stages was conducted (Appendix S1 and S2). Table 6 shows the results of the sensitivity test. Among oral cancer patients who underwent only surgery, we presumed that staging distribution in model A was the same in different age group (<45 years and >45 years) as that from the Bureau of Health Promotion of around 83% was early stage (AJCC stage I & II). It reveals that elder patients (>45 years) undergoing surgery alone had a 1.52-fold risk of mortality (95% CI, 1.15-2.01). In model B, we hypothesized that younger patients (<45 years) had 100% of early stage oral cancer. After adjusting other factors, elder patients (>45 years) still showed a 1.46-fold risk of mortality (95% CI, 1.10-1.95). Among oral cancer patients who underwent surgery with adjuvant therapy, the staging distribution in model A was assumed in the same way that near 77% patients were advanced stage (AJCC stage III & IV) in different age group. In model B, we hypothesized that elder patients (>45 years) had 100% of advanced stage oral cancer. The adjusted hazard ratios for death in both models disclose no statistically elevated among younger patients [HR, 1.16 (95% CI, 0.96-1.40); HR, 0.95 (95% CI, 0.67-1.36)].

**Table 6:** The adjusted hazard ratios of provider category in different regression model (n=2339) ***.

Variable	Event/total (%)	Model A*			Model B**	
		HR	95%CI		HR	95%CI
Surgery						
Age<45	66/304(22)	1			1	
Age≧45	285/967(3)	1.52	(1.15-2.01)		1.46	(1.10-1.95)
Surgery + adjuvant therapy						
Age<45	160/304(53)	1			1	
Ageγ45	435/764(57)	1.16	(0.96-1.40)		0.95	(0.67-1.36)

Abbreviation: HR, hazard ration; 95%CI, 95% confidence interval

* Adjusted for patients’ age, gender, Charlson Comorbidity Index Score, primary site, surgeon caseload within two years, hospital level, socioeconomic status, region of residence, and urbanization.

** Adjusted for patients’ age, gender, Charlson Comorbidity Index Score, primary site, surgeon caseload within two years, hospital level, socioeconomic status, region of residence, urbanization, and simulation stage.

*** Please see the Appendix S1 and Appendix S2 for the distribution of cancer stages in different simulation models

In summary, oral cancer patients with young age (<45 years) didn’t confer a worse survival rates. The result was robust as the survival rates were determined using the Cox proportional regression model, stratification by propensity scores and sensitivity test.

## Discussion

Oral cancer is a disease of middle-aged and old men who use tobacco, alcohol, and betel nut. An increasing incidence rate of oral cancer has been noted in younger patients. The primary objective of this study was to compare the survival rates of oral cancer patients younger than 45 with those of patients older than 45. After adjustment for the patients’ gender, primary tumor site, Charlson Comrobidity Index score, treatment modality, surgeon caseload, hospital teaching level, enrollee category, level of urbanization, and geographic region, the hazard ratio for death was 1.8-times (*P*<0.001) greater for the oldest group (>65 years) than for the youngest group (<45 years). This negative association remained statistically significant by using propensity score for analysis. Sensitivity test using simulation stage also revealed no elevated risk of mortality among younger patients (<45 years).

The literature concerning prognosis for young patients with oral cancer is conflicting. Some studies concluded that the disease was more aggressive in younger patients [3-5]. Some studies revealed that younger patients had a better survival rate [11-15]; however, other studies did not show a significant difference between the different age groups [6-10]. A common problem in these studies was a small sample size. Using a population-based database, our study provided strong evidence to support the proposition that young oral cancer patients did not have a worse prognosis. It has been suggested that oral cancer in young patients is a different entity. Ligen et al. found that increased p53 expression without mutation in exon 5-9 was noted in squamous cell carcinoma of the oral cavity in young, non-smoking patients [32]. Schantz et al. reported greater chromosome fragility in lymphocytes from young patients with head and neck cancer following belomycin treatment [33]. Other authors have reported that cyclin D1 gene polymorphism (CCND1) was associated with the early onset of head and neck cancer, and contributed to susceptibility to head and neck cancer, particularly in young non-smokers and non-drinkers in a case-control study [34]. Oral mucosa is similar to genital mucosa. The susceptibility of both mucosae to herpes simplex virus (HSV) and human papilloma virus (HPV) has suggested that HSV and HPV play a role in the cause of oral cancer. Parkin et al. and zur Hausen reported that up to 25% of oral cancer is associated with HPV infection [1,35] Hafkamp et al. [36] suggested that HPV is more commonly detected in young patients with head and neck cancer and it has been related to down-regulation of pRb, overexpression of p16^INK4A^ and wild-type p53. Kassim and Daley reported that HSV-1 has a direct relationship with oral squamous cell carcinoma, but its role in cellular transformation is not clear [37].

The quality of the risk-adjustment technique used in analyzing administrative information is an important issue. In the first part of our study, the Cox proportional regression model was used to validate the effect of young age versus middle and old age. We found a significantly increased adjusted hazard ratio for oral cancer patients in the old age group. Old age (>65 years) patients were found to have a 83% higher risk of death (*P*<0.001) after adjusting for comorbid conditions and other confounding factors. However, there were differences with regard to age, gender, tumor site and clinical condition between different age groups and the results of the Cox proportional regression model could be challenged by others. In the second part of our series, propensity scores were used to stratify the patients into five groups with similar propensity scores in order to reduce the effects of selection bias between the different age groups [28,29,38]. Oral cancer patients with young age (<45 years) did not have a higher risk of mortality, compared to middle age (45-65 years) or old age (>65 years) patients. Difference of carcinogenesis in oral cancer between young age and old age patients may explain some of results we observed.

Cancer stage is an important factor for long-term survival in oral cancer, but it is not available in our database. However, oral cancer stage is largely related to primary tumor size, metastatic lymph node site and number, and it causes whether surgery alone or surgery with adjuvant chemo-radiotherapy will be performed. We applied another national database to gain the distribution of oral cancer stage under different treatment modalities (surgery or surgery with adjuvant therapy) in Taiwan. We further performed a sensitivity test with simulation cancer stage. Among oral cancer patients who underwent only surgery, we set the number of early stage (AJCC stage I & II) patients in the younger group (<45 years) in two different models which were 83% (around nationwide percentage) and 100%. Similarly, among oral cancer patients undergoing surgery with adjuvant therapy, advanced stage (AJCC III & IV) patients in the elder group (>45 years) in two different models were set as 77% (around nationwide percentage) and 100%. Even in the most impossible scenario (all younger patients undergoing surgery alone were belonged to early stage oral cancer or all elder patients undergoing surgery with adjuvant therapy were belonged to advanced stage oral cancer), younger group (<45 years) remained no elevated risk of mortality.

Our study has several limitations. First, it is lack of access to detailed information from the insurance claims database with regard to oral cancer stage, which is the important variable of the survival. However, we performed sensitivity test by using simulation cancer stage and the results were consistent. Further study is indicated using cancer registry data with more details on staging. Second, the database lacks information of lifestyle factors such as dietary habits, alcohol, betel nut or tobacco use, which may be risk factors and prognostic factors for oral cancer [39]. Third, oral cancer patients who received only resection were not included, so that the interpretation of these results is limited to oral cancer patients who received resection and reconstruction. However, given the robust magnitude of the effects and statistical significance of the effects in this study, these limitations are unlikely to compromise our results.

In summary, our findings showed the effect of age on the rate of survival for oral cancer patients in a population-based study. For those oral cancer patients who underwent wide excision and reconstruction with or without adjuvant therapy, young age did not confer a worse prognosis using the Cox proportional regression model, propensity score and sensitivity test. Further research is necessary to investigate the etiology and molecular markers for oral cancer in young patients. Young patients with oral cancer may be treated using general guidelines and there may be no need for more aggressive treatment.

## Supporting Information

Appendix S1 **Distribution of cancer stage among oral cancer patients with surgery alone in different simulation models.**
(DOC)

Appendix S2 **Distribution of cancer stage among oral cancer patients with surgery and adjuvant therapy in different simulation models.**
(DOC)
