# Supplementary material for: Impact of Young Age on the Prognosis for Oral Cancer: A Population-Based Study in Taiwan
Source: PLoS One. 2013 Sep 26;8(9):e75855. doi: 10.1371/journal.pone.0075855 (PMC3784390; doi:10.1371/journal.pone.0075855)
Supplement: Appendix S1 — Distribution of cancer stage among oral cancer patients with surgery alone in different simulation models. (DOC) [file pone.0075855.s001.doc]

| Model A |  | Early stage | | Advanced stage | |  |  |
| --- | --- | --- | --- | --- | --- | --- | --- |
| Age<45 | (n=304) | 261 | (83%) | 43 | (17%) |  | 304 |
| Age≧45 | (n=967) | 832 | (83%) | 135 | (17%) |  | 967 |
|  |  |  |  |  |  |  |  |

Appendix S1. Distribution of cancer stage among oral cancer patients with surgery alone in different simulation models.

| Model B |  | Early stage | | Advanced stage | |  |  |
| --- | --- | --- | --- | --- | --- | --- | --- |
| Age<45 | (n=304) | 304 | (100%) | 0 | (0%) |  | 304 |
| Age≧45 | (n=967) | 789 | (82%) | 178 | (18%) |  | 967 |
|  |  |  |  |  |  |  |  |
