# Supplementary material for: Impact of Young Age on the Prognosis for Oral Cancer: A Population-Based Study in Taiwan
Source: PLoS One. 2013 Sep 26;8(9):e75855. doi: 10.1371/journal.pone.0075855 (PMC3784390; doi:10.1371/journal.pone.0075855)
Supplement: Appendix S2 — Distribution of cancer stage among oral cancer patients with surgery and adjuvant therapy in different simulation models. (DOC) [file pone.0075855.s002.doc]

Appendix S2. Distribution of cancer stage among oral cancer patients with surgery and adjuvant therapy in different simulation models.

| Model A |  | Early stage | | Advanced stage | |  |  |
| --- | --- | --- | --- | --- | --- | --- | --- |
| Age<45 | (n=304) | 70 | (23%) | 234 | (77%) |  | 304 |
| Age≧45 | (n=764) | 176 | (23%) | 588 | (77%) |  | 764 |
|  |  |  |  |  |  |  |  |

| Model B |  | Early stage | | Advanced stage | |  |  |
| --- | --- | --- | --- | --- | --- | --- | --- |
| Age<45 | (n=304) | 246 | (81%) | 58 | (19%) |  | 304 |
| Age≧45 | (n=764) | 0 | (0%) | 764 | (100%) |  | 764 |
|  |  |  |  |  |  |  |  |
